# Effectiveness of Health Education on Dietary Habits and Oral Hygiene Practices: An Interventional Study

**DOI:** 10.7759/cureus.94620

**Published:** 2025-10-15

**Authors:** Sindhu Ravindra, Faika Kolkar, Sushmini Hegde, Archana Krishnamurthy

**Affiliations:** 1 Oral Medicine and Radiology, The Oxford Dental College and Hospital, Bengaluru, IND; 2 Public Health Dentistry, The Oxford Dental College and Hospital, Bengaluru, IND

**Keywords:** diet, nutrition, oral health, oral hygiene practices, patient education

## Abstract

Background: Dietary habits significantly influence oral health, yet patient awareness of this relationship remains inadequate. This study aimed to assess and improve knowledge and practices regarding the influence of diet on oral health among outpatients.

Methods: A pre-post-test interventional study was conducted among 100 patients (53 males, 47 females). Data on demographic characteristics, oral hygiene practices, and knowledge/attitudes toward diet-related oral health were collected using a structured questionnaire. An educational intervention was administered, followed by a post-test after a defined period. Descriptive statistics and paired t-tests were used to analyze changes.

Results: Pre-intervention, 73% brushed twice daily or more, 65% used fluoridated toothpaste, and 57% recognized the role of diet in oral health. Awareness of specific dietary recommendations, such as the benefits of high-fiber foods over fruit juice (26%) and risks of frequent snacking (42%), was low. Post-intervention, significant improvements were observed in oral hygiene practices (mean score: 4.12 ± 1.60 to 8.86 ± 1.40, p-value < 0.001) and dietary knowledge/attitudes (mean score: 4.03 ± 2.52 to 9.56 ± 1.23, p-value < 0.001). Brushing twice daily increased to 92%, optimal toothpaste use to 97%, and awareness of the role of diet in oral health to 92%. Recognition of the benefits of high-fiber foods rose to 98%, and awareness of sugar sources increased to 93%.

Conclusion: The intervention significantly enhanced both oral hygiene practices and knowledge regarding the influence of diet on oral health. These findings highlight the value of targeted patient education in improving preventive oral health behaviors.

## Introduction

Nutrition plays a crucial role in maintaining oral health, while oral health, in turn, influences dietary habits and nutritional status, affecting the overall health outcomes and quality of life [1]. Similarly, oral health is an essential component of the dental care system, and understanding the dietary factors that affect it is a key consideration in developing effective treatment plans. Dietary factors, particularly sugar and other cariogenic nutrients, exert a substantial impact on dental and periodontal tissues. Dietary patterns are therefore an important consideration in oral healthcare, as they contribute to the development of conditions such as oral mucosal lesions, tooth loss, periodontal disease, and dental caries. Moreover, poor oral health has been associated with systemic conditions, including pulmonary infections, cardiovascular disease, diabetes mellitus, and respiratory disorders. The relationship between nutrition and dental caries is well established: specific oral bacteria ferment dietary sugars under anaerobic conditions, producing organic acids that demineralize enamel and dentin [2].

The prevention of oral diseases is greatly aided by nutrition education, which should be considered an integral component of dental and oral health management plans. Such education enhances treatment effectiveness, supports the maintenance of good oral health, and reduces the risk of developing new dental problems. Higher nutritional knowledge is associated with improved dietary habits and better nutritional status, underscoring the importance of understanding the dietary factors that influence oral health. Oral diseases are among the most prevalent noncommunicable diseases (NCDs) worldwide, affecting an estimated 3.58 billion individuals, nearly half of the global population, and representing a significant public health concern in both developed and developing countries.

Dental caries is a multifactorial condition driven by dysbiosis of the oral microbiome, involving various cariogenic species such as *mutans streptococci *(MS), *Lactobacilli*, *Scardovia wiggsiae*, and several *Actinomyces* species, all of which share acid production and acid tolerance as cariogenic traits [3]. Based on their effects on teeth, food items are classified as cariogenic, cariostatic, or anticariogenic [4]. The type of sugar also influences caries risk; for instance, lactose has a smaller cariogenic effect than other sugars, while xylitol has demonstrated the ability to inhibit bacterial growth and reduce caries incidence. Beverages such as soft drinks, sweetened coffee and tea, and acidic foods and candies promote tooth demineralization and decay, while fruit juices, often containing extrinsic sugars and low pH, contribute to erosive tooth wear [5]. Evidence suggests that fresh fruits are preferable to fruit juices, as chewing stimulates saliva production and enhances its cleansing effect. Meal frequency also plays a role, with fewer meals per day being associated with lower caries risk, and consuming desserts with meals being preferable to between-meal snacking [6].

A thorough literature search has revealed limited focused research on the knowledge and awareness of nutritional aspects affecting oral health among the public or dental patients [6]. This gap underscores the need for studies that evaluate awareness levels and inform targeted educational strategies aimed at improving dietary practices and oral health outcomes. Accordingly, the present study was undertaken to assess the level of awareness regarding the influence of diet on oral health among patients, thereby addressing this gap and contributing to the understanding of nutrition-related oral health knowledge.

## Materials and methods

The study was conducted among patients attending the Outpatient Department of Oral Medicine and Radiology at the Oxford Dental College and Hospital, Bengaluru, India, from 15 March 2024 to 15 March 2025. Being a new questionnaire-based study, a convenience sample of 100 patients was selected. Patients aged 18 years and above, who were comfortable with reading and conversing in the English language, were included in the study. Patients not willing to be a part of the study were excluded. Informed consent was obtained from all participants prior to enrolment.

A 20-item, closed-ended, self-constructed questionnaire was developed in accordance with the guidelines of the WHO Oral Health Survey 2013 (fifth edition) by a panel of five members, including oral health experts and one lay representative [7]. To assess internal consistency, the questionnaire was piloted on 20 patients, and Cronbach’s alpha was calculated. With a reliability value exceeding 0.8, the questionnaire was finalized and subsequently distributed among the study participants. The responses from the initial 20 patients were excluded from the final analysis, which was conducted on a sample of 100 patients (see Appendix A).

Demographic data, along with information on participants’ knowledge, practices, and dietary patterns related to oral health, were collected during the pre-test phase, which was allotted 10 minutes for completion. Oral hygiene practice scores and knowledge and attitude scores were calculated as the sum of correct responses, with one point assigned for each correct answer and zero for incorrect answers. No negative marking was applied. For interpretation, the total scores were converted into percentages of the maximum achievable score and categorized as follows: scores ≤33% were considered poor, 34-66% as fair, and ≥67% as good.

Following the pre-test, participants were provided a standardized 15-minute PowerPoint presentation (Microsoft® Corp., Redmond, WA, USA) on oral health education was delivered to the participants. The session incorporated flow charts to illustrate key concepts and short educational videos to reinforce proper dietary and oral hygiene practices, thereby enhancing participant understanding and engagement. The session was designed and validated by the expert panel to ensure accuracy and comprehensibility, and covered key domains including the importance of oral hygiene in overall health, the impact of diet on oral health, recommended daily hygiene practices such as toothbrushing and flossing, recognition and prevention of common oral diseases, and the role of regular dental visits and preventive measures. The presentation also highlighted the adverse effects of tobacco, alcohol, and betel nut use on oral and systemic health. Simple language, illustrative visuals, and interactive discussion points were incorporated to enhance participant engagement and retention. Two weeks after the intervention, the same questionnaire was re-administered as a post-test to assess changes in oral health knowledge and practices.

Completed questionnaires were collected, coded, and entered into a database for analysis. Statistical analysis was performed using IBM SPSS Statistics for Windows, Version 23 (IBM Corp., Armonk, NY, USA). Descriptive statistics, including frequencies and percentages, were used to summarize the distribution of responses to each questionnaire item in the pre- and post-test phases, allowing evaluation of changes in oral health knowledge and practices. Mean scores before and after the intervention were compared using the paired t-test, and results are presented as mean ± standard deviation. A p-value <0.05 was considered statistically significant.

## Results

The study comprised 53 male and 47 female participants. The age distribution and their education level are presented in Table 1.

**Table 1:** Demographic characteristics of the study population N: number of subjects; %: percentage

Characteristic		N	%
Age group in years	≤25	48	48.0
	26-45	46	46.0
	≥46	6	6.0
Educational level	Illiterate	0	0
	Primary school	1	1.0
	Middle school	0	0
	High school	16	16.0
	Diploma	31	31.0
	Graduate and postgraduate	43	43.0
	Professional	9	9.0

The pre-test revealed that 73% of respondents cleaned their teeth twice or more daily, while 27% did so less frequently. Almost all participants (98%) used a toothbrush, with 65% using fluoridated toothpaste and 35% using non-fluoridated variants. Regarding toothpaste quantity, 37% used the optimal amount, whereas 63% used less than recommended. Only 30% reported brushing for more than two minutes, while the majority (70%) brushed for less than two minutes. In terms of other oral hygiene practices, 42% of respondents cleaned their tongue using a tongue cleaner, whereas 58% either used alternative methods or did not clean their tongue at all. Rinsing the mouth after eating was reported by 33% of participants, while 67% did not practice rinsing. Dental service utilization was generally low: 84% of respondents had not visited a dentist in the past six months. Among those who visited, 95% did so due to a specific complaint. Sources of oral health information varied, with 87% receiving information from non-dental sources and only 13% obtaining it directly from a dentist (Table 2).

**Table 2:** Pre-test oral hygiene practices among the respondents N: number of subjects; %: percentage

Question no.	Oral hygiene practice	Yes		No	
		N	%	N	%
1.	Clean teeth once or more a day	73	73	27	27
2.	Use toothbrush to clean the teeth	98	98	2	2
3.	Use fluoridated toothpaste to clean the teeth	65	65	35	35
4.	Use optimal amount of toothpaste	37	37	63	63
5.	Brush teeth for more than two minutes	30	30	70	70
6.	Clean the tongue	42	42	58	58
7.	Rinse mouth after eating food	33	33	67	67
8.	Seen a dentist in less than six months	16	16	84	84
9.	Last visited a dentist with a complaint	5	5	95	95
10.	Get oral health information from many sources	13	13	87	87

Regarding knowledge of diet and nutrition in relation to oral health, 57% of respondents believed that food plays an important role in maintaining oral health. Awareness of specific dietary recommendations was generally low. Only 26% knew that consuming high-fiber foods was better for oral health than drinking fruit juice, and 72% were unaware that common dietary sources of sugar include soft drinks, candies, and cookies. Similarly, only 23% knew that eating sweets as part of a meal is preferable to consuming them separately, and 58% recognized that snacking throughout the day is more harmful to teeth than eating foods as part of main meals. Knowledge about the relationship between food and oral disease mechanisms was also limited. Sixty percent of participants did not know that food trapped between teeth can lead to tooth decay, and 62% were unaware that vitamin deficiencies can cause gum disease. Less than half (48%) knew that sticky foods increase the risk of tooth decay. While 58% understood that extrinsic acids in drinks can erode teeth, 41% did not know that spicy foods may cause oral mucosal lesions (Table 3). The mean score for oral hygiene habits was 4.12 ± 1.6, while knowledge and attitude towards oral hygiene practices showed a mean score of 4.03 ± 2.52.

**Table 3:** Pre-test distribution of respondents according to knowledge and attitude on dietary habits N: number of subjects; %: percentage

Question no.	Knowledge and attitude on dietary habits	Yes		No	
		N	%	N	%
11.	Food plays an important role in oral health	57	57	43	43
12.	Eating high fibre fresh fruit is better than fruits juice	26	26	74	74
13.	Common sources of sugar in the diet include soft drinks, candy, cookies, and pastries	28	28	72	72
14.	Eating sweets with meals is better than eating sweets between meals to prevent tooth decay	23	23	77	77
15.	Foods that are eaten as part of a meal cause less harm to teeth and tissue in mouth than eating lots of snacks throughout the day	42	42	58	58
16.	Food stuck in the teeth and between the teeth are associated with tooth decay and gum disease	40	40	60	60
17.	If your diet lacks certain nutrients such as vitamin C, vitamin A, folate, it may be more difficult for tissues in your mouth to resist infection. This may contribute to gum disease.	38	38	62	62
18.	Sticky sweets increase the probability of tooth decay compared to non-sticky sweets	48	48	52	52
19.	Extrinsic acids such as citric, phosphoric, ascorbic that are found in fruit juices, in drinks, and in vinegar cause corroding of the teeth.	42	42	58	58
20.	Spicy food increases the probability of oral mucosal lesion compared to less spicy food	59	59	41	41

Post-test findings indicated marked improvements in oral hygiene practices. The proportion of respondents cleaning their teeth more than once daily increased to 92%. The use of a toothbrush remained high at 95%, representing a slight 3% decrease from pre-test levels. The proportion using fluoridated toothpaste rose substantially to 97%, while 97% reported using the optimal amount of toothpaste. Brushing for two minutes or more increased to 92%, and 89% of respondents reported cleaning their tongue with a tongue cleaner. Rinsing the mouth after eating was reported by 91% of participants. Dental service utilization and information sources also showed improvement. Eighty-one percent of respondents reported visiting a dentist for an oral health check-up in the past six months, compared to a much lower pre-test proportion. Among them, 76% visited due to a specific complaint. The proportion receiving oral health information directly from a dentist rose to 76%, while 24% reported obtaining information from other sources, such as mobile applications (Table 4).

**Table 4:** Post-test oral hygiene practices among the respondents N: number of subjects; %: percentage

Question no.	Oral hygiene practice	Yes		No	
		N	%	N	%
1.	Clean teeth once or more a day	92	92	8	8
2.	Use toothbrush to clean the teeth	95	95	5	5
3.	Use fluoridated toothpaste to clean the teeth	97	97	3	3
4.	Use optimal amount of toothpaste	97	97	3	3
5.	Brush teeth for than two minutes	92	92	8	8
6.	Cleaning tongue with tongue cleaner	89	89	11	11
7.	Rinse mouth after eating food	91	91	9	9
8.	Seen a dentist in less than six months	81	81	19	19
9.	Last visited a dentist with a complaint	76	76	24	24
10.	Get oral health information from many sources	76	76	24	24

Post-intervention, knowledge and attitudes regarding dietary habits related to oral health showed substantial improvement. The proportion of respondents who believed that food plays an important role in oral health increased to 92%. Almost all participants (98%) recognized that consuming high-fiber foods is better for oral health than drinking fruit juice, and only 7% remained unaware that common dietary sources of sugar include soft drinks, candies, and cookies. Awareness that eating sweets as part of a meal is preferable to consuming them separately rose to 96%, and 94% understood that snacking throughout the day is more harmful than eating foods as part of main meals. Knowledge of diet-disease mechanisms also improved markedly. Only 4% of participants were unaware that food trapped between teeth can cause decay, and just 3% did not know that vitamin deficiencies can lead to gum disease. Similarly, only 4% were unaware that sticky foods increase the risk of tooth decay. Almost all respondents (96%) recognized that extrinsic acids in drinks can erode teeth, and only 2% remained unaware that spicy foods may cause oral mucosal lesions (Table 5). The mean score for oral hygiene habits increased to 8.86 ± 1.4, while knowledge and attitudes towards oral hygiene practices improved to a mean score of 9.56 ± 1.23.

**Table 5:** Post-test distribution of respondents according to knowledge and attitude on dietary habits N: number of subjects; %: percentage

Question no.	Knowledge and attitude on dietary habits	Yes		No	
		N	%	N	%
11.	Food plays an important role in oral health	92	92	8	8
12.	Eating high fibre fresh fruit is better than fruits juice	98	98	2	2
13.	Common sources of sugar in the diet include soft drinks, candy, cookies, and pastries	93	93	7	7
14.	Eating sweets with meals is better than eating sweets between meals to prevent tooth decay	96	96	4	4
15.	Foods that are eaten as part of a meal cause less harm to teeth and tissue in mouth than eating lots of snacks throughout the day	94	94	6	6
16.	Food stuck in the teeth and between the teeth are associated with tooth decay and gum disease	96	96	4	4
17.	If your diet lacks certain nutrients such as vitamin C, vitamin A, folate, it may be more difficult for tissues in your mouth to resist infection. This may contribute to gum disease.	97	97	3	3
18.	Sticky sweets increase the probability of tooth decay compared to non-sticky sweets	96	96	4	4
19.	Extrinsic acids such as citric, phosphoric, ascorbic that are found in fruit juices, in drinks, and in vinegar cause corroding of the teeth.	96	96	4	4
20.	Spicy food increases the probability of oral mucosal lesion compared to less spicy food	98	98	2	2

The mean score for oral hygiene practices increased significantly from 4.12 ± 1.60 pre-intervention to 8.86 ± 1.40 post-intervention (p < 0.001). Likewise, knowledge and attitudes regarding dietary habits improved markedly, with mean scores rising from 4.03 ± 2.52 to 9.56 ± 1.23, a difference that was also statistically significant (p < 0.001) (Table 6).

**Table 6:** Comparison of pre-test and post-test scores ^#^ Paired t-test was used to compare the means; * Statistically highly significant

	Pre-test, mean score	Post-test, mean score	t-value^#^	P-value
Oral hygiene habits	4.12 ± 1.6	8.86 ± 1.4	22.251	<0.001*
Knowledge and attitude	4.03 ± 2.52	9.56 ± 1.23	18.497	<0.001*

## Discussion

This study assessed the awareness of the impact of diet on oral health among patients aged over 18 years in Bengaluru city. Participants demonstrated an overall average level of awareness regarding dietary influences on oral health and general oral hygiene practices, with the findings indicating insufficient knowledge in both domains. Dietary practices, particularly the consumption of free sugars, are well-established risk factors for NCDs [8]. The association between dietary proteins and dental caries has also been recognized since the 1950s [9]. This study is distinct in simultaneously evaluating diet-related knowledge and broader oral health attitudes and practices, with emphasis on hygiene behaviors [10].

Several factors influence individual food choices, including lack of awareness, availability, cost, and financial constraints [11]. In this study, socio-demographic variables such as age, gender, education, employment, and income were analyzed, with socioeconomic status showing a significant association with oral diet knowledge.

The overall low mean scores mirror the findings from previous research. Bapat et al. reported poor dental nutrition knowledge among nutrition and dietetics students in Udaipur, India, while Paulo et al. found that 80% of surgeons lacked confidence in providing nutritional guidance [6,12]. Similar gaps have been observed internationally. Szatko et al. noted inadequate dietary awareness among Polish mothers, Jordanian adults showed limited knowledge of periodontal disease prevention, and Badrasawi et al. identified insufficient nutrition knowledge among dental patients [9,13,14].

In this study, general oral health questions were more frequently answered correctly than diet-specific items. Basic concepts, such as the role of sugar in dental caries, were well understood, similar to findings among Saudi Arabian school teachers and school children [9]. However, more specialized items, such as the effects of sugar frequency, sweetener types, snacking habits, sticky foods, and protective dietary factors like dairy products or nuts, were poorly answered, consistent with earlier studies [6].

No significant gender-based differences in knowledge were observed, despite previous reports often noting higher nutritional awareness among females [8,13,14]. Comparable results have been reported in Malaysia, where no gender difference was found in oral health awareness [11]. The present findings highlight persistent gaps in both general and diet-specific oral health knowledge, underscoring the need for targeted educational interventions that address both basic and advanced nutrition concepts relevant to oral health.

Despite the valuable insights provided, this study was limited by its conduct in a single geographical location, which restricts the generalizability of the findings to the broader national population. Furthermore, the offline survey was completed only by literate individuals who visited the outpatient department and were sufficiently interested in the subject, thereby introducing potential selection bias. This study has certain limitations. It lacked a control group, which restricts the ability to establish causal relationships. The follow-up period was relatively short, which could have also led to recall bias, leading to exceptionally high post-intervention improvements. Thus, studies with longer follow-up duration and repeated assessments are required.

## Conclusions

The findings of this study highlight an overall insufficient awareness regarding the relationship between diet and oral health among adults in Bengaluru, with notable gaps in knowledge related to specific dietary factors influencing oral health. While general oral health concepts were relatively well understood, diet-specific knowledge, particularly regarding the effects of sugar frequency, protective dietary components, and the role of various food types, remained limited. These results underscore the need for targeted educational interventions that address both general and specialized aspects of diet-related oral health. Incorporating nutrition-focused oral health education into community outreach, dental consultations, and public health programs may help bridge these knowledge gaps and promote healthier dietary practices.

## Appendices

Appendix A

Survey Questionnaire

Demographic data

Name:

Age/Gender:

Educational level:

Occupation:

Monthly income:

Questionnaire on Oral Health Practices

(Put tick mark for the selection option)

1.     How often do you clean your teeth?

Never............................................................................... ☐

Once a month.................................................................. ☐

2-3 times a month........................................................... ☐

Once a week.................................................................... ☐

2-6 times a week............................................................. ☐

Once or more a day......................................................... ☐

2.     Do you use any of the following to clean your teeth?

Toothbrush................................................................ ☐

Wooden toothpicks .................................................... ☐

Thread (dental floss) ................................................. ☐

Charcoal or any other.......................................... ☐

Chewstick/miswak...................................................... ☐

3.     Do you use toothpaste to clean your teeth? Yes ☐        No ☐

4.  Do you use a toothpaste that contains fluoride? Yes ☐    No ☐

5.     How much toothpaste do you use for brushing?

Pea size………..☐

Half-length of brush……..☐

Whole length of brush…..☐

6.     How long do you clean your teeth??

Less than a minute………☐

1 min ………………......☐

2 min……………………☐

More than two min………☐

7.     How do you clean your tongue??

Hand ………….☐

Tongue cleaner……☐

Tooth brush…………☐

Do not clean the tongue…..☐

8.     Do you rinse your mouth after eating food??

Yes………..☐

No………..☐

Occasionally…….☐

9.     How long was it since you last saw a dentist?

Less than 6 months......................................................... ☐

6-12 months.................................................................... ☐

More than 1 year but less than 2 years........................... ☐

2 years or more but less than 5 years.............................. ☐

5 years or more/never ..................................................... ☐

10.  What was the reason of your last visit to the dentist?

Consultation/advice......................................................... ☐

Pain or trouble with teeth, gums or mouth..................... ☐

Treatment/ follow-up treatment....................................... ☐

Routine check-up/treatment............................................ ☐

Don’t know/don’t remember........................................... ☐

11.  What is source of your oral health information??

 Tv……………… ☐

Health magazines……………..☐

Mobile………..☐

Dentist …☐

Questionnaire on dietary habits

(Put tick mark for the selection option)

1.     Food plays an important role in oral health

True……………… ☐

False ……………..☐

I do not know……☐

2.     Eating high fiber Fresh fruit is good the fruits juice??

True……………… ☐

False ……………..☐

I do not know……☐

3.     Common sources of sugar in the diet includes soft drinks, candy, cookies, and pastries??

True……………… ☐

False ……………..☐

I do not know……☐

4.     Eating sweets with meals is better than eating sweets between meals to prevent tooth decay

True……………… ☐

False ……………..☐

I do not know……☐

5.     Foods that are eaten as part of a meal cause less harm to teeth and tissue in mouth than eating lots of snacks throughout the day.

True……………… ☐

False ……………..☐

I do not know……☐

6.     Food stuck in the teeth and between the teeth are associated with tooth decay and gum disease

True……………. ☐

False …………….☐

I do not know……☐

7.     If your diet lacks certain nutrients such as vitamin C, vitamin A, folate, it may be more difficult for tissues in your mouth to resist infection. This may contribute to gum disease.

True……………… ☐

False ……………..☐

I do not know……☐

8.     Sticky sweets increase the probability of tooth decay compared to non sticky sweets

True……………… ☐

False ……………..☐

I do not know……☐

9.     Extrinsic acids such as citric, phosphoric, ascorbic that are found in fruit juices, in drinks, and in vinegar cause corroding of the teeth.

True……………… ☐

False ……………..☐

I do not know……☐

10.  Spicy food increases the probability of oral mucosal lesion compared to less spicy food.

True……………… ☐

False ……………..☐

I do not know……☐

## References

[1] Haq MW, Tanwir F, Nawaz M (2015). Association of systemic diseases on tooth loss and oral health. J Biomed Sci.

[2] Scardina GA, Messina P (2012). Good oral health and diet. J Biomed Biotechnol.

[3] Zhan L (2018). Rebalancing the caries microbiome dysbiosis: targeted treatment and sugar alcohols. Adv Dent Res.

[4] Mahan KL (2016). Krause's Food & the Nutrition Care Process. https://shop.elsevier.com/books/krauses-food-and-the-nutrition-care-process/mahan/978-0-323-34075-5.

[5] Hujoel PP, Lingström P (2017). Nutrition, dental caries and periodontal disease: a narrative review. J Clin Periodontol.

[6] Bapat S, Asawa K, Bhat N (2016). Assessment of dental nutrition knowledge among nutrition/dietetics students. J Clin Diagn Res.

[7] (2013). Oral Health Surveys: Basic Methods - 5th Edition. World Health Organization.

[8] Lin S (2018). The Dental Diet: The Surprising Link between Your Teeth, Real Food, and Life-Changing Natural Health. https://www.hayhouse.co.in/books/the-dental-diet/.

[9] Badrasawi MM, Hijjeh NH, Amer RS, Allan RM, Altamimi M (2020). Nutrition awareness and oral health among dental patients in Palestine: a cross-sectional study. Int J Dent.

[10] El-Qaderi SS, Taani DQ (2004). Oral health knowledge and dental health practices among schoolchildren in Jerash district/Jordan. Int J Dent Hyg.

[11] Muyide AM, Oduneye MT (2021). Effect of dietary pattern and nutritional status on oral health of patients attending the dental clinic in University College Hospital, Ibadan, Nigeria. Int J Health Sci Res.

[12] Paulo DA, de Oliveira BM, Wang DW, Guimarães MP, Cukier C, Lopes Filho Gde J (2013). Surgeons' knowledge and attitude regarding concepts of nutritional therapy [Article in Portuguese]. Rev Col Bras Cir.

[13] Szatko F, Wierzbicka M, Dybizbanska E, Struzycka I, Iwanicka-Frankowska E (2004). Oral health of Polish three-year-olds and mothers’ oral health-related knowledge. Community Dent Health.

[14] Taani DQ (2002). Periodontal awareness and knowledge, and pattern of dental attendance among adults in Jordan. Int Dent J.

